# Divergent Organism-Specific Trajectories of Multidrug Resistance Across the COVID-19 Pandemic: An Interrupted Time-Series Study in a Mexican Tertiary Hospital, 2017–Early 2026

**DOI:** 10.3390/antibiotics15101005

**Published:** 2026-10-10

**Authors:** Jesús García-Gómez, Mauricio Alfredo Ambriz-Alarcón, Brian Rafael Rubio-Mora, Luis Felipe Jave-Suárez, Adriana Aguilar-Lemarroy, María Martha Villaseñor-García, Georgina Hernández-Flores, María Concepción Rodríguez-Lora, Teresita De Jesús Hernández-Flores, Liliana Iñiguez-Gutiérrez, Luis Eduardo del Moral-Trinidad, Anibal Samael Méndez-Clemente

**Affiliations:** 1 División de Inmunología, Centro de Investigación Biomédica de Occidente (CIBO), Instituto Mexicano del Seguro Social (IMSS), Sierra Mojada 800, Colonia Independencia, Guadalajara 44340, Jalisco, Mexico; jesus.garcia9891@alumnos.udg.mx (J.G.-G.); luis.jave@academicos.udg.mx (L.F.J.-S.); mvillasr@gmail.com (M.M.V.-G.); georgina.hernandezf@imss.gob.mx (G.H.-F.); 2 Programa de Doctorado en Ciencias en Biomédicas, Centro Universitario de Ciencias de la Salud (CUCS), Universidad de Guadalajara, Guadalajara 44340, Jalisco, Mexico; 3 División de Medicina, Antiguo Hospital Civil de Guadalajara “Fray Antonio Alcalde”, Calle Hospital 278, Centro Barranquitas, Guadalajara 44280, Jalisco, Mexico; mambriz@hcg.gob.mx; 4 Urgencias Adultos, Hospital Civil de Guadalajara “Fray Antonio Alcalde”, Calle Hospital 278, Centro Barranquitas, Guadalajara 44340, Jalisco, Mexico; brubio@hcg.gob.mx; 5 Unidad de Investigación Epidemiológica y en Servicios de Salud, Instituto Mexicano del Seguro Social (IMSS), Guadalajara 44340, Jalisco, Mexico; adriana.aguilar@academicos.udg.mx; 6 Laboratorio de Microbiología, Antiguo Hospital Civil de Guadalajara “Fray Antonio Alcalde”, Calle Hospital 278, Centro Barranquitas, Guadalajara 44280, Jalisco, Mexico; conchisrl14@hotmail.com; 7 Departamento de Disciplinas Filosófico, Metodológicas e Instrumentales, Instituto de Investigación de Recursos Humanos en Salud, Centro Universitario de Ciencias de la Salud, Universidad de Guadalajara, Guadalajara 44340, Jalisco, Mexico; teresita.hflores@academicos.udg.mx; 8 Departamento de Disciplinas Especializadas I, Decanato de Ciencias de la Salud, Universidad Autónoma de Guadalajara, Av. Patria 1201, Guadalajara 45129, Jalisco, Mexico; liliana.gutierrez@edu.uag.mx; 9 Unidad de Vigilancia Epidemiológica y Medicina Preventiva, Antiguo Hospital Civil de Guadalajara “Fray Antonio Alcalde”, Calle Hospital 278, Centro Barranquitas, Guadalajara 44280, Jalisco, Mexico

**Keywords:** antimicrobial resistance, multidrug-resistant pathogens, segmented regression, interrupted time series, COVID-19

## Abstract

**Background/Objectives**: Whether the COVID-19 pandemic uniformly increased antimicrobial resistance (AMR) is contested, and aggregate analyses may mask organism-specific patterns. The objective of the study was to characterize changes in the level and slope of monthly multidrug-resistant (MDR), possible extensively drug-resistant (pXDR), possible pandrug-resistant (pPDR), vancomycin-resistant enterococci (VRE), and methicillin-resistant *Staphylococcus aureus* (MRSA) positivity at a Mexican tertiary-care hospital, and to assess heterogeneity by organism, antimicrobial class, specimen type, and hospital service. **Methods**: Retrospective interrupted time-series of deduplicated bacterial isolates (October 2017 to January 2026), analyzed by segmented regression across pre-pandemic, pandemic, and post-peak phases. **Results**: Cohort-wide MDR positivity had been declining before 2020 and rose abruptly at pandemic onset (level shift +6.81 percentage points, *p* = 0.0092) with a simultaneous slope reversal, reaching 24.27 percentage points above the extrapolated pre-pandemic trend by February 2022 (95% CI 14.26–34.28, *p* < 0.0001). The decline resumed after the pandemic peak, but the two slope changes did not offset one another (joint *p* = 0.0064). pXDR and pPDR moved in the same direction (+8.93, *p* = 0.0233; +15.40, *p* = 0.0113). VRE showed no significant departure, whereas MRSA declined in every phase yet departed significantly from its extrapolated trajectory (+23.68, *p* = 0.0040). Among species, *Escherichia coli* showed the largest departure from its counterfactual (+48.82 percentage points, *p* < 0.0001) and *Klebsiella pneumoniae* had the largest abrupt level rise (+14.46 percentage points, *p* = 0.0055), while *S. aureus* declined in every phase despite an abrupt rise in level at pandemic onset. At the class level, carbapenem and third-/fourth-generation cephalosporin non-susceptibility rose at pandemic onset and returned to baseline afterwards (19.1% to 28.0% to 18.7%, and 51.7% to 59.4% to 52.5%; all q < 0.001), whereas fluoroquinolone non-susceptibility rose and did not revert, climbing further in *E. coli* (61.5% to 65.2% to 72.7%, q = 0.0012) and plateauing at an elevated level in *K. pneumoniae* (37.6% to 46.5% to 49.3%, q = 0.1919 post-peak). **Conclusions**: Resistance trajectories across pandemic-related phases were divergent and, for the Gram-negative organisms, incompletely reversed, with the highest burden of advanced resistance in high-intensity settings. Organism-resolved surveillance and sustained stewardship are needed.

## 1. Introduction

Antimicrobial resistance (AMR) is a leading threat to global health. In 2019, it was associated with an estimated 4.95 million deaths worldwide, of which 1.27 million were directly attributable [1], and the attributable burden is projected to reach approximately 1.91 million deaths annually by 2050, with Latin America and the Caribbean among the most affected regions [2]. In the Americas, 569,000 deaths were associated with bacterial AMR in 2019, and 141,000 were directly attributable, unevenly distributed among pathogens; *Escherichia coli*, *Staphylococcus aureus*, *Klebsiella pneumoniae*, *Pseudomonas aeruginosa*, and *Acinetobacter baumannii* contributed most, reflecting their clinical frequency, hospital-acquired infection transmissibility, and capacity to accumulate resistance determinants [1,3].

The 2024 WHO Bacterial Priority Pathogens List placed carbapenem-resistant *A. baumannii* and Enterobacterales in the critical category, alongside carbapenem-resistant *P. aeruginosa*, methicillin-resistant *S. aureus* (MRSA), and vancomycin-resistant *Enterococcus faecium* as major priorities [4,5]. Several belong to the ESKAPE group (*Enterococcus faecium*, *Staphylococcus aureus*, *Klebsiella pneumoniae*, *Acinetobacter baumannii*, *Pseudomonas aeruginosa* and *Enterobacter* spp.), whose success reflects intrinsic resistance, mobile genetic elements, genomic plasticity, and environmental persistence [6]. Because *Escherichia coli* rivals or exceeds these organisms in clinical frequency and in attributable mortality, it is increasingly grouped with them as ESKAPE-E, and we adopt that grouping here. Standardized multidrug-resistant (MDR), extensively drug-resistant (XDR), and pandrug-resistant (PDR) definitions improve comparability but depend on the organism-specific panel tested and on breakpoint standards, so a pathogen may show a high MDR proportion without a comparable XDR or PDR burden [7]. These distinctions are particularly relevant in intensive care, where Gram-negative organisms predominate among culture-positive infections and selected resistant phenotypes are associated with higher mortality [8].

The COVID-19 pandemic disrupted this landscape. Antibiotics were prescribed to 74.6% of patients with COVID-19, whereas bacterial co-infection was documented in only 8.6% [9], a prescribing-need mismatch echoed across co-infection reviews [10,11]. Opposing pressures could raise or lower resistance, broad-spectrum use, prolonged intensive care unit (ICU) stay, invasive devices and interrupted stewardship on one side against masking, hand hygiene, and visitor restrictions on the other, so that the net direction depends on which set dominated in a given hospital [12]. Empirical evidence reflects this heterogeneity: a meta-analysis of pandemic-associated resistance rates found no significant overall change in MRSA or vancomycin-resistant enterococci and only heterogeneous signals for Gram-negative organisms [13], while a separate systematic review reported that resistance was nonetheless common among COVID-19 patients with documented bacterial infections, although susceptibility data were reported for at least one species in only 63% of the included studies and between-study heterogeneity was substantial [14]. National U.S. surveillance reported a combined 20% rise in seven hospital-onset resistant infections, peaking in 2021, yet trajectories differed by pathogen, with hospital-onset MRSA the exception: it remained essentially stable and was the only one of the seven to decline from 2021 to 2022 [15]. *A. baumannii*, favored by environmental persistence and clonal spread [16], roughly doubled during the pandemic in one ICU cohort [17], but this cannot be extrapolated to other organisms.

In Mexico, phenotypic surveillance had already documented high and heterogeneous resistance before COVID-19, including extended-spectrum β-lactamase-producing *E. coli* and *K. pneumoniae* and carbapenem resistance in non-fermenting Gram-negative bacilli [18,19], an elevated baseline that any pandemic-associated change must be read against. Pandemic-era data likewise showed divergence: *A. baumannii* resistance increased, while *K. pneumoniae* remained unchanged and quinolone resistance declined in *E. coli* and *P. aeruginosa* [20]. Most studies, however, relied on simple pre-pandemic versus pandemic comparisons over short periods, which cannot separate an abrupt level change from continuation of a pre-existing trend. Aggregate estimates are weighted by isolate volume and therefore follow the most frequently isolated organisms, so that a less common organism carrying a disproportionate share of extensively or pandrug-resistant isolates leaves the cohort figure largely unchanged.

We therefore analyzed deduplicated bacterial isolates from a tertiary-care referral hospital in Guadalajara, México (October 2017 to January 2026), using segmented interrupted time-series regression to assess changes in the level and slope of monthly MDR, possible XDR (pXDR), possible PDR (pPDR), VRE, and MRSA proportions across three pandemic-related phases, with secondary analyses by organism, antimicrobial class, specimen type, and hospital service. We hypothesized divergent rather than uniform trajectories, with the greatest burden of advanced resistance occurring in hospital-adapted pathogens and high-intensity clinical settings.

## 2. Results

### 2.1. Organism Distribution and Resistance Patterns

**Figure 1:**
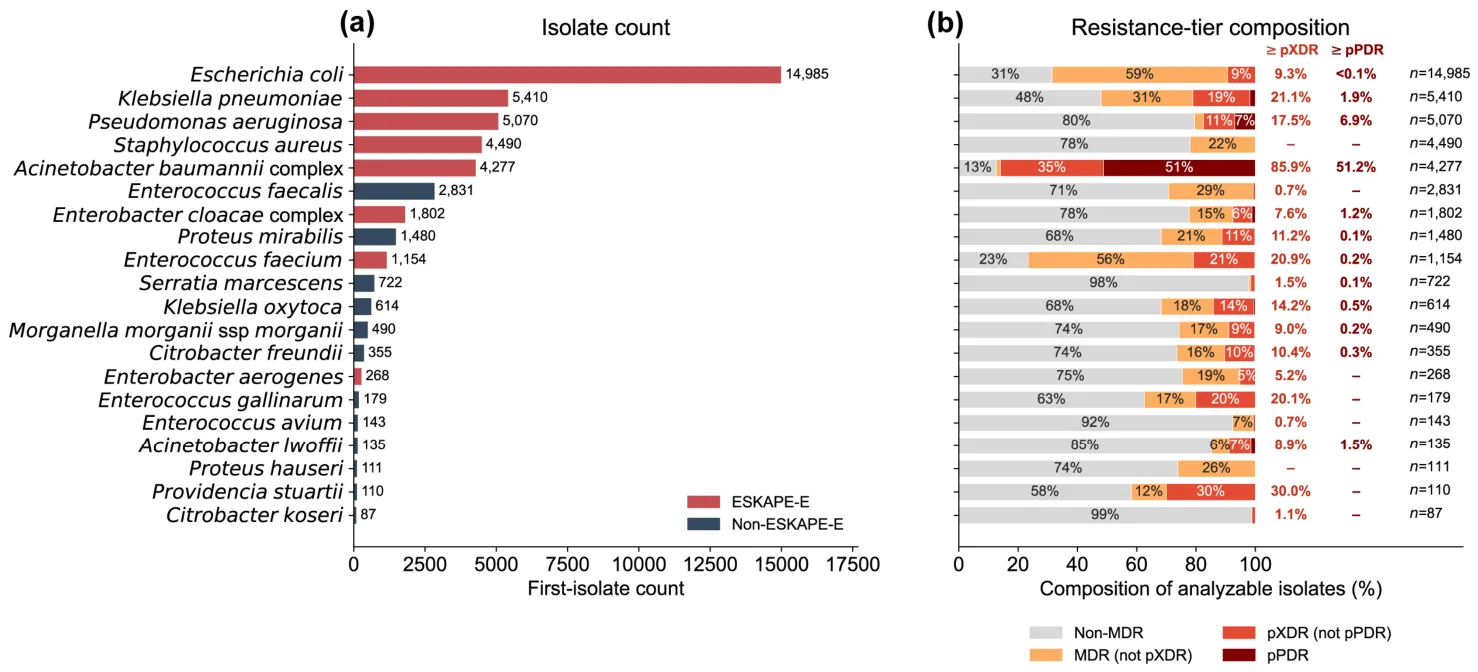
Isolate counts and resistance-tier composition by organism. (**a**) Total first-isolate counts for the 20 most frequently recovered organisms, colored by ESKAPE-E status. (**b**) Resistance-tier composition among evaluable isolates as mutually exclusive categories (pPDR > pXDR > MDR > non-MDR); categories sum to 100% per organism; n indicates the evaluable denominator.

Across the study period, *Escherichia coli* was by far the most frequent organism (14,985 first isolates), followed by *Klebsiella pneumoniae* (5410), *Pseudomonas aeruginosa* (5070), *Staphylococcus aureus* (4490), and the *Acinetobacter baumannii* complex (4277), with several belonging to the ESKAPE-E group (Figure 1a). Figure 1 is restricted to organisms for which the Magiorakos criteria define an applicable-category set; coagulase-negative staphylococci, the most frequent organisms falling outside those criteria, are consequently not shown. Resistance-tier composition varied markedly by organism (Figure 1b): the *A. baumannii* complex carried by far the highest advanced-resistance burden (85.9% pXDR or higher, of which 51.2% pPDR), whereas *E. coli*, despite its dominance, combined a high MDR proportion (68.5%) with little advanced resistance (9.3% pXDR or higher, <0.1% pPDR); *K. pneumoniae* was intermediate (52.0% MDR, 21.1% pXDR or higher).

**Figure 2:**
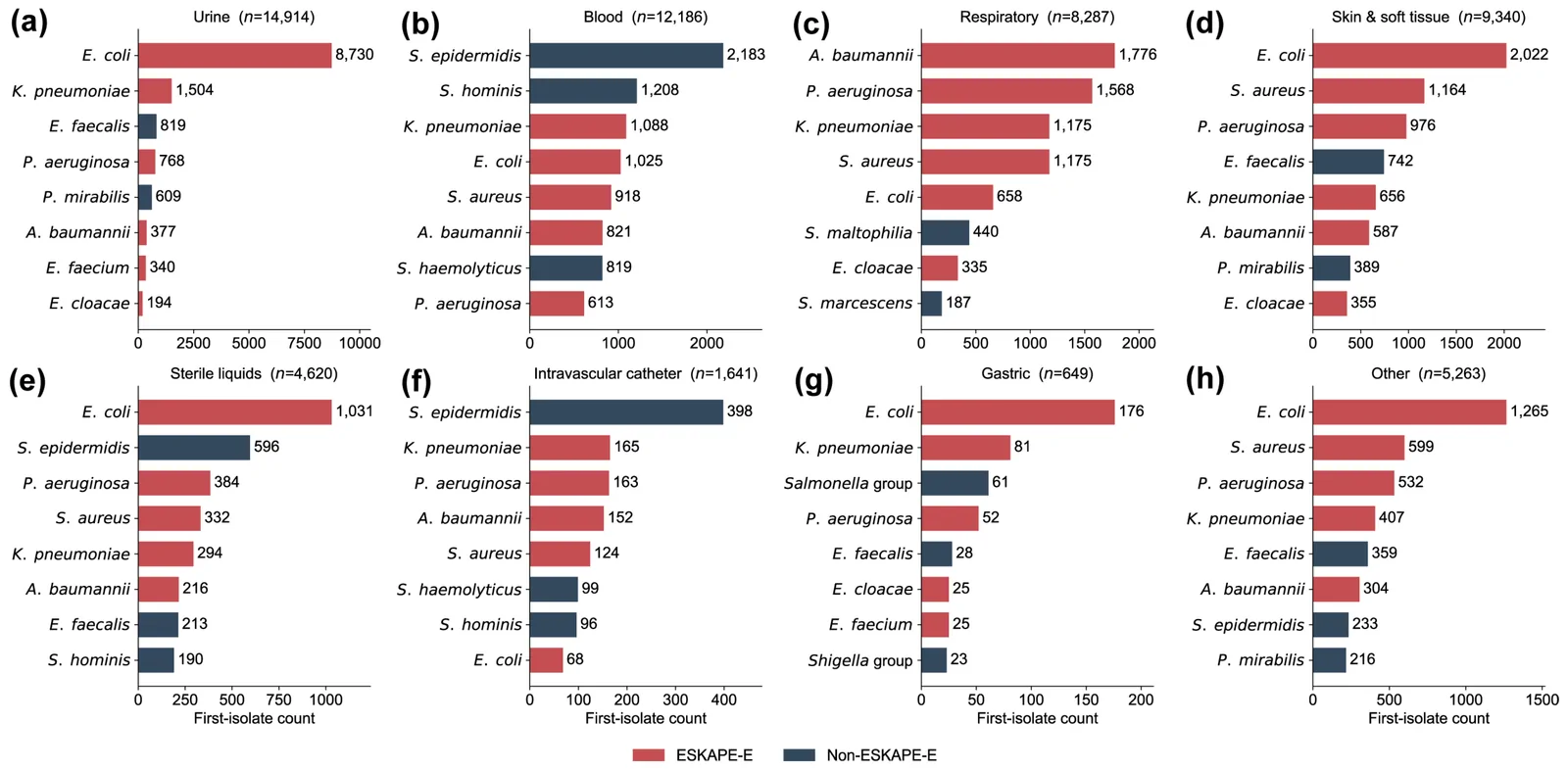
Top eight bacterial organisms by clinical specimen type. First-isolate counts for the eight most frequently recovered organisms across eight specimen categories: (**a**) urine, (**b**) blood, (**c**) respiratory, (**d**) skin and soft tissue, (**e**) sterile fluids, (**f**) intravascular catheter, (**g**) gastric, (**h**) other. Bars colored by ESKAPE-E status; *n* indicates total first isolates per specimen.

Organism composition also differed by specimen (Figure 2): *E. coli* led urine (8730/14,914), skin and soft tissue, and gastric and “other” specimens; blood and intravascular-catheter specimens were led by *S. epidermidis*; and respiratory specimens were led by the ESKAPE-E organisms (*A. baumannii complex* (1776), *P. aeruginosa* (1568)).

**Figure 3:**
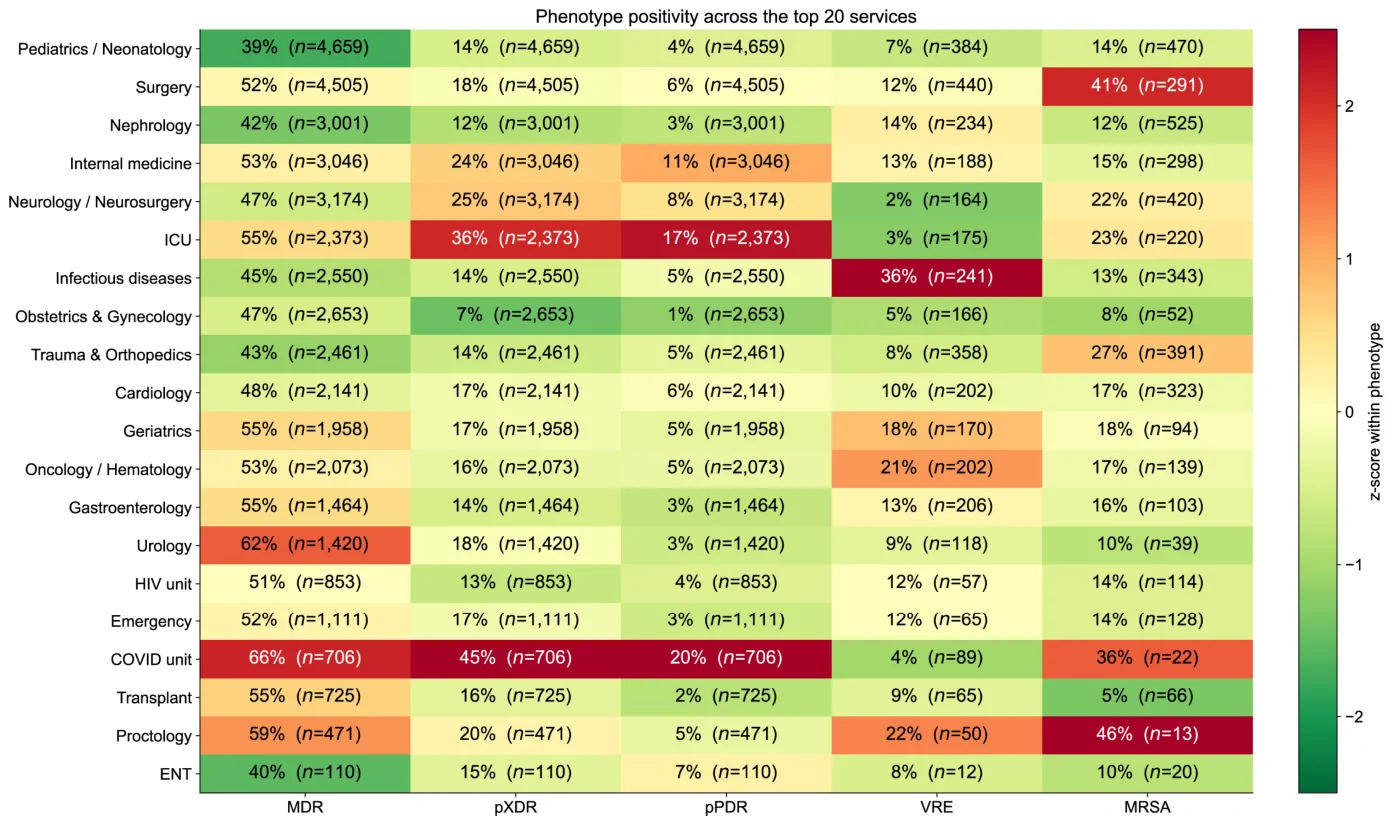
Resistance phenotype positivity across the 20 highest-volume clinical services. Heatmap of MDR, pXDR, pPDR, VRE, and MRSA (%) by service, color-scaled as a within-phenotype z-score across services; cell values show raw positivity; n is the evaluable denominator.

Across the 20 highest-volume services (Figure 3), the COVID unit carried the highest positivity for all three multidrug-resistant tiers (MDR 65.7%, pXDR 44.6%, pPDR 20.3%; *n* = 706), followed by the ICU (54.7%, 35.7%, 16.9%; *n* = 2373). The gradient was clearest for the advanced tiers; high MDR positivity also occurred in lower-acuity services (Urology 62.3%, *n* = 1420). VRE positivity was highest in Infectious Diseases (35.7% of 241 enterococcal isolates) and MRSA was highest in Surgery (41.2% of 291 *S. aureus* isolates); the numerically highest MRSA value, in Proctology, rested on 13 isolates and is not interpreted.

### 2.2. Cohort-Wide Temporal Trends

**Figure 4:**
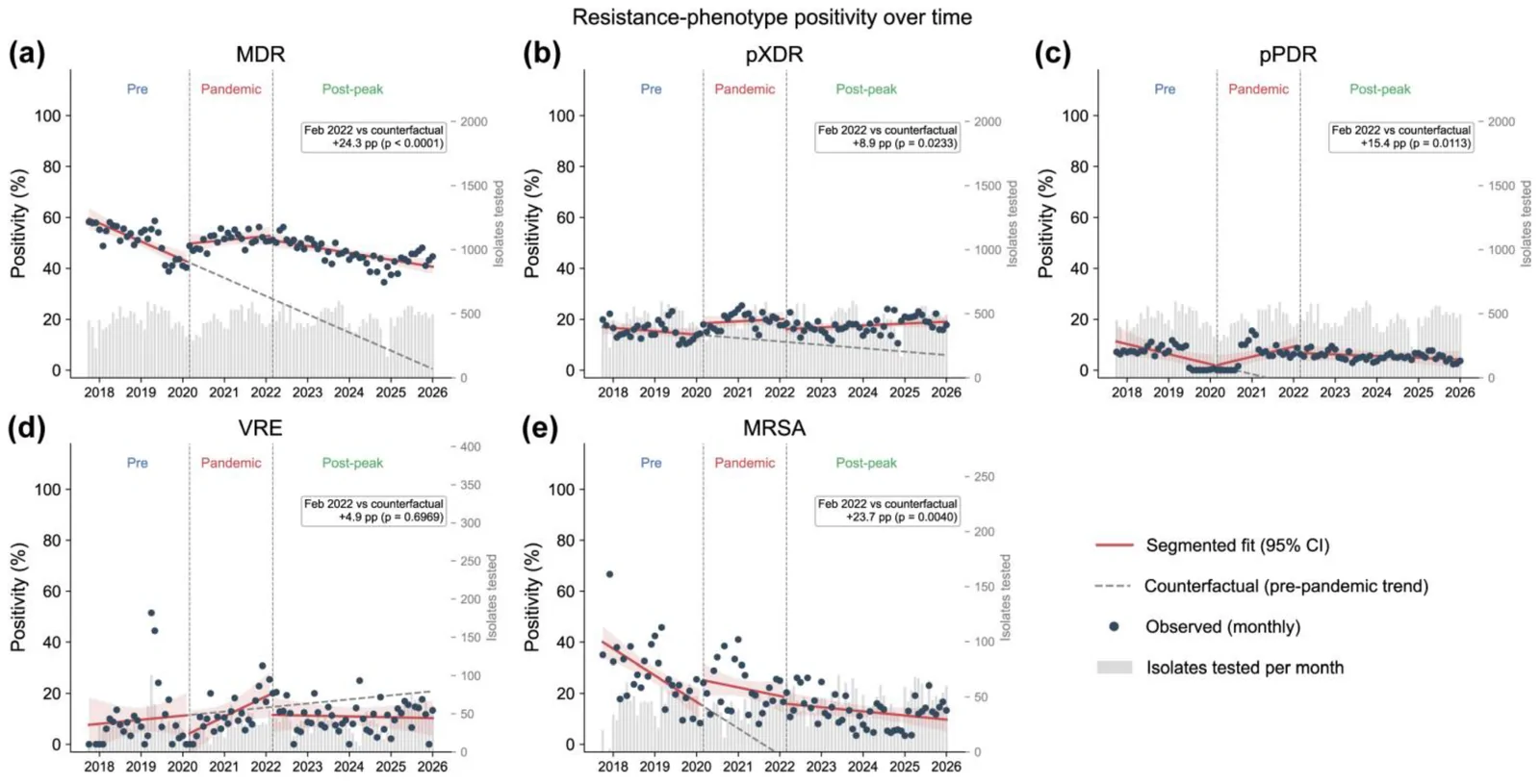
(**a**) MDR, (**b**) pXDR, (**c**) pPDR, (**d**) VRE, (**e**) MRSA. Monthly observed positivity (points), segmented regression fit with 95% CI (line and band) and pre-pandemic counterfactual (dashed line); phase labels mark the three phases; grey bars show monthly isolates tested. Annotation reports the February 2022 counterfactual contrast and *p*-value.

Segmented regression was applied to MDR, pXDR, pPDR, VRE, and MRSA (Table 1, Figure 4). For MDR (fitted by generalized least squares with an AR(1) term owing to residual autocorrelation, Durbin–Watson 1.23), baseline positivity was 60.09% and declined before the pandemic (β_1_ = −0.60 percentage points (pp)/month, *p* < 0.0001). At pandemic onset, positivity rose abruptly (β_2_ = +6.81 pp, *p* = 0.0092), and the decline was arrested (β3 = +0.73 pp/month, *p* = 0.0003; net pandemic slope +0.13 pp/month, *p* = 0.3488). After the pandemic peak, the decline resumed at a shallower rate than before 2020 (β_5_ = −0.36 pp/month, *p* = 0.0220; net post-peak slope −0.23 pp/month, *p* = 0.0001), with no significant level shift at that boundary (β_4_ = −1.54 pp, *p* = 0.5050). The joint test of the two slope changes was significant (*p* = 0.0064). Both slope changes held under Newey–West standard errors. The fitted level exceeded the extrapolated pre-pandemic trend by 24.27 pp in February 2022 (95% CI 14.26–34.28, *p* < 0.0001) and by 40.14 pp in January 2026 (95% CI 19.64–60.63, *p* = 0.0002).

The more severe tiers moved in the same direction by the end of the pandemic phase: pXDR exceeded its extrapolated trend by 8.93 pp (95% CI 1.24–16.62, *p* = 0.0233), and pPDR exceeded it by 15.40 pp (95% CI 3.58–27.23, *p* = 0.0113) (Figure 4b,c; Table A2 and Table A3). The two single-genus phenotypes diverged: VRE showed no significant departure (+4.89 pp, *p* = 0.6969), whereas MRSA departed significantly (+23.68 pp, *p* = 0.0040), while its net slope remained negative in every phase (−0.87, −0.28 and −0.14 pp/month) (Figure 4d,e; Table A4 and Table A5). Sensitivity analyses covering interruption dates, panel coverage, model specification, and case mix are reported in Tables S1–S6.

### 2.3. Species-Level MDR

**Figure 5:**
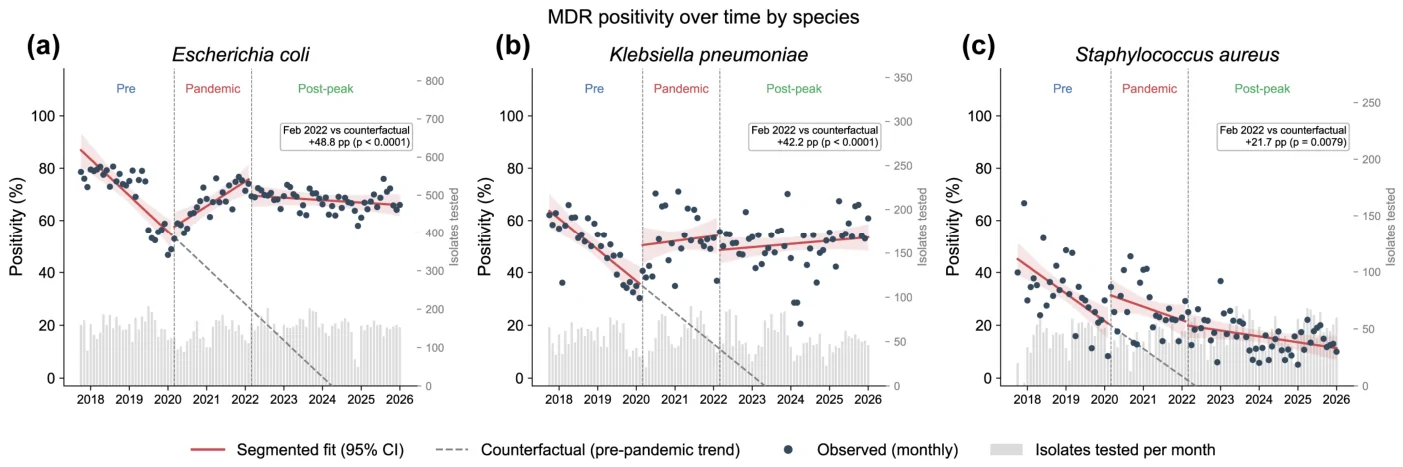
MDR positivity over time by species. (**a**) *Escherichia coli*, (**b**) *Klebsiella pneumoniae*, (**c**) *Staphylococcus aureus*. Elements as in Figure 4.

Because the cohort-wide trajectory can mask species differences, *E. coli*, *K. pneumoniae,* and *S. aureus* were modeled individually (Figure 5). All three departed significantly from their extrapolated pre-pandemic trajectories, but through different mechanisms.

*E. coli* (Table 2) had baseline of 88.03% and the steepest pre-pandemic decline of the three (β_1_ = −1.15 pp/month, *p* < 0.0001). At pandemic onset, the slope reversed (β_3_ = +1.94 pp/month, *p* < 0.0001) to a significantly positive net pandemic slope (+0.79 pp/month, *p* = 0.0002), without an abrupt change in level (β_2_ = +2.24 pp, *p* = 0.5370). The post-peak level shift was not significant in the primary model (β_4_ = −6.11 pp, *p* = 0.0659), although it was under Newey–West standard errors (*p* < 0.0001), and the post-peak net slope was flat (−0.08 pp/month, *p* = 0.3356). The joint test of the two slope changes was significant (*p* < 0.0001); the counterfactual difference was +48.82 pp in February 2022 (95% CI 33.75–63.88, *p* < 0.0001); the January 2026 value (+93.23 pp) is not reported as supporting because the pre-pandemic slope extrapolates below zero before that point.

*K. pneumoniae* (Table 3) differed in mechanism: baseline 64.68%, a pre-pandemic decline of −1.00 pp/month (*p* < 0.0001; ordinary least squares, Durbin–Watson 1.64), and an abrupt level rise at pandemic onset (β_2_ = +14.46 pp, *p* = 0.0055) alongside a slope change (β_3_ = +1.16 pp/month, *p* = 0.0009), with neither post-peak term significant (β_4_, *p* = 0.2137; β_5_, *p* = 0.8496). Its net pandemic and post-peak slopes did not differ from zero (+0.16 pp/month, *p* = 0.5530; +0.11 pp/month, *p* = 0.2840). The joint test was significant (*p* < 0.0001), and the counterfactual difference was +42.21 pp in February 2022 (95% CI 24.93–59.49, *p* < 0.0001); the January 2026 value (+88.31 pp) is not reported as supporting because the pre-pandemic slope extrapolates below zero before that point.

*S. aureus* (Table A6) declined before the pandemic (β_1_ = −0.87 pp/month, *p* < 0.0001), and its net slope remained negative in every phase (−0.87, −0.43 and −0.19 pp/month; *p* = 0.0800 and *p* = 0.0380 for the pandemic and post-peak phases). It nonetheless showed an abrupt level rise at pandemic onset (β_2_ = +11.02 pp, *p* = 0.0178) and a significant counterfactual difference (+21.66 pp, 95% CI 5.82–37.51, *p* = 0.0079; joint test *p* = 0.0015).

### 2.4. Antibiotic-Class Non-Susceptibility

**Figure 6:**
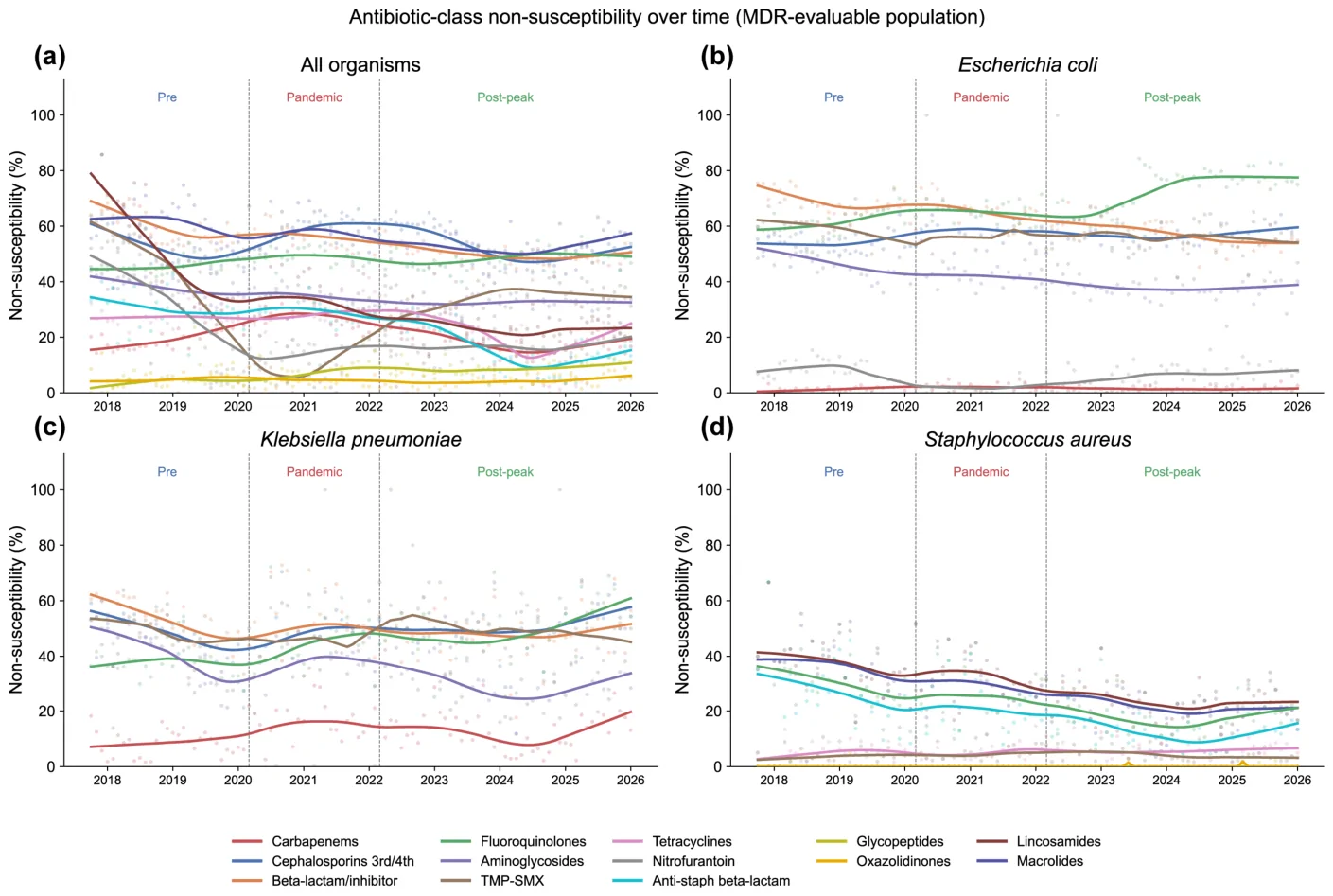
Antibiotic-class non-susceptibility over time in the MDR-evaluable population. (**a**) all organisms, (**b**) *Escherichia coli*, (**c**) *Klebsiella pneumoniae*, (**d**) *Staphylococcus aureus*. LOESS-smoothed monthly non-susceptibility (%) per antibiotic class, restricted to classes with ≥200 isolates tested and ≥24 months with ≥20 tested; points show monthly values; phase labels as in Methods. TMP-SMX, trimethoprim-sulfamethoxazole.

Class-level non-susceptibility clarified which classes underlay these shifts (Figure 6, Table 4). Across all organisms, carbapenem non-susceptibility rose at pandemic onset and returned to baseline afterwards (19.1% pre-pandemic, 28.0% pandemic, 18.7% post-peak; *q* < 0.0001 for both transitions). Third-/fourth-generation cephalosporin non-susceptibility followed the same shape with smaller amplitude (51.7% to 59.4%, 52.5%; *q* = 0.0007 and *q* = 0.0005). Fluoroquinolone non-susceptibility did not revert: in *E. coli*, it rose at pandemic onset and again afterwards (61.5% to 65.2%, 72.7%, *q* = 0.0073 and *q* = 0.0012), whereas in *K. pneumoniae*, it rose and then plateaued (37.6% to 46.5%, 49.3; *q* = 0.0034 and *q* = 0.1919). *S. aureus* showed the opposite pattern, with no pandemic-onset rise in either class and declines that reached significance only afterwards (fluoroquinolones 29.6%, 25.9%, 18.1%; *q* = 0.1920 and *q* = 0.0014; anti-staphylococcal β-lactams 25.6%, 21.5%, 12.9%; *q* = 0.1603, *q* = 0.0004). Because these comparisons treat consecutive monthly proportions as independent observations, they are reported as descriptive rather than as tests of interruption.

Because the ciprofloxacin breakpoint was revised during the study period, the direction of the post-peak fluoroquinolone trend depends on which criterion is applied throughout: among Enterobacterales, ciprofloxacin non-susceptibility across the three phases was 45.9%, 49.0%, and 56.7% as reported, 50.2%, 49.0%, and 56.7% under the current breakpoint, and 42.1%, 41.2%, and 39.9% under the earlier one (Table S5). The composite MDR counterfactual for the same organisms was robust to this manipulation (+35.3, +33.9 and +38.8 pp under the three criteria, each *p* < 0.0001; Table S6).

## 3. Discussion

In this near-decade-long interrupted time series from a Mexican tertiary-care referral hospital, the COVID-19 pandemic was not associated with a uniform rise in antimicrobial resistance but with organism- and phenotype-specific trajectories. Cohort-wide MDR positivity, which had been declining before 2020, rose abruptly at pandemic onset and its decline was arrested. That decline resumed after the pandemic peak but at a shallower rate, and the two slope changes did not offset one another (joint *p* = 0.0064). The fitted level exceeded the extrapolated pre-pandemic trend by 24.27 percentage points in February 2022. The more severe pXDR and pPDR tiers moved in the same direction, whereas the two single-genus phenotypes diverged: VRE showed no significant pandemic departure, while MRSA departed significantly from its pre-pandemic trajectory in the primary model even though its positivity continued to fall in every phase. At the class level, carbapenem and third-/fourth-generation cephalosporin non-susceptibility rose and returned to baseline with the pandemic phase, whereas fluoroquinolone non-susceptibility in *E. coli* and *K. pneumoniae* did not revert. Together, these patterns were consistent with our a priori hypothesis of divergent rather than convergent resistance dynamics.

These findings help reconcile apparently conflicting reports. Aggregate national surveillance, such as the U.S. analysis of seven hospital-onset resistant infections, documented a combined ~20% pandemic increase that peaked in 2021 [15], whereas systematic reviews found no consistent overall effect for MRSA or vancomycin-resistant enterococci and only heterogeneous signals among Gram-negative organisms [13]. Our data show that both observations can hold within a single institution: a real, Gram-negative-dominated increase that did not fully reverse, superimposed on divergent organism-specific baselines. Our VRE result is consistent with the pooled estimate; MRSA is where we differ, departing significantly from its pre-pandemic trajectory while continuing to decline in absolute terms. That combination is invisible to a pre-versus-post comparison, and separating it is what segmented regression is for: distinguishing an abrupt pandemic-associated change from the continuation of a pre-existing trend.

The organism-resolved results are the most informative. *E. coli* showed the largest departure from its extrapolated pre-pandemic trajectory, driven entirely by a slope reversal rather than an abrupt change in level, and it had not returned to that trajectory by 2026. *K. pneumoniae* differed in form rather than in magnitude, with an abrupt level rise at pandemic onset together with a slope change that never reverted. This contrasts with an earlier Mexican tertiary-care series in which *K. pneumoniae* resistance appeared stable during 2021–2022 [20], underscoring how sensitive such conclusions are to the observation window and analytic method. *S. aureus*, by contrast, continued to decline in every phase, although it too showed an abrupt rise in level at pandemic onset. Consistent with its recognized capacity for environmental persistence and clonal nosocomial spread [16,17], the *A. baumannii* complex carried the highest combined pXDR/pPDR burden of any organism, and the COVID and ICU unit—setting in which mechanical ventilation, invasive devices and patient cohorting are common—carried the highest pXDR and pPDR positivity, in keeping with the exposure-related epidemiology described in large ICU cohorts [8].

A plausible contributor to the reverting component of these class-level signals is the well-documented surge in antibiotic exposure during the early pandemic [21]. In México, this pattern is quantifiable: in a national IMSS cohort of 214,171 adult COVID-19 inpatients, 25.3% received empirical antibiotics, of which cephalosporins accounted for 75.7% [22]; and in a Mexican secondary-care hospital, total antibiotic consumption peaked in 2020 at 330 defined daily doses (DDD)/100 bed days, dominated by WHO Watch-group agents (83.1%), before falling by roughly half in 2021–2022 [23]. Community prescribing followed suit, with ceftriaxone (25%), azithromycin (19%), and clarithromycin among the most prescribed drugs in México City outpatients and 28% of prescriptions self-initiated [24]. The temporal correspondence is notable, since carbapenem and cephalosporin non-susceptibility rose and returned to baseline over the same interval in which that consumption peak was reported and then receded, whereas fluoroquinolone non-susceptibility did not return. Part of that difference is methodological rather than biological, since the ciprofloxacin breakpoint was revised during the study period and the post-peak fluoroquinolone trend is sensitive to which criterion is applied throughout (Table S5).

The concordance is in any case ecological and hypothesis-generating rather than causal, and no inference about individual-level exposure and resistance follows from it; the documented decrease in consumption at a Mexican pediatric center over the same period [25] further cautions against assuming uniform national exposure and mirrors the phenotype-specific heterogeneity in our data.

These observations carry policy relevance. México has progressively strengthened antimicrobial governance: the 2010 restriction of over-the-counter antibiotic sales coincided with a fall in national consumption from 10.5 to 7.5 DDD/1000 inhabitants/day [26], and the Estrategia Nacional de Acción contra la Resistencia a los Antimicrobianos, made mandatory in 2018, aligned national policy with the WHO Global Action Plan under a One Health framework [27,28]. Also, data from the PUCRA network, which include Jalisco, showed a considerable increase in hospital antibiotic consumption over the pandemic period: the median consumption of cephalosporins, the most frequently used class across participating hospitals, rose from 17.8 DDD per 100 bed days in 2020 to a peak of 27 in 2022 and stayed elevated thereafter (24.48 in 2024) [29]. This trend parallels the rise in cephalosporin non-susceptibility we observed at pandemic onset, although our institutional non-susceptibility returned to baseline after 2022, while regional consumption continued to rise. Analyses such as the present one illustrate the granularity that organism-resolved surveillance within such networks can add to aggregate benchmarking. However, implementation of hospital antimicrobial stewardship remains uneven across México [30], and stewardship activities were widely disrupted during the pandemic even as empirical prescribing rose [31]. Reinforcing stewardship and infection prevention during future surges could blunt comparable increases, since structured hospital stewardship interventions improve prescribing and reduce resistant infections without compromising clinical outcomes [32]. Metrics such as the WHO AWaRe classification [33], under which Watch-group agents dominated early pandemic consumption [23], offer a practical stewardship target for the recovery period.

The principal strengths of this study are a long, continuous series spanning distinct pre-pandemic, pandemic, and post-peak phases, a large isolate volume, and an analytic approach that separates levels from slope change and resolves trajectories by organism, antimicrobial class, specimen, and service rather than reporting a single aggregated outcome. Several limitations should nonetheless be considered. This was a retrospective, single-center study at a tertiary-level referral hospital, and its case-mix limits generalizability to other settings; whether the trajectories described here are institution-specific or reflect broader regional patterns will require multicenter surveillance using comparable phase definitions. Resistance phenotypes were derived from routine antibiogram results rather than molecular confirmation, so a phenotype is not equated with a resistance mechanism. Because routine panels were often incomplete, the extensively and pandrug-resistant tiers are reported as possible rather than confirmed, and incomplete panels bias the tiers in opposite directions: MDR requires non-susceptibility in at least three categories and is therefore harder to meet when fewer categories are tested, whereas pXDR and pPDR are defined relative to the categories that were evaluable and are correspondingly easier to meet. Both the number of categories tested and the agents within them varied over the study period (Table S2), although restricting the cohort to isolates with progressively broader panel coverage did not abolish the cohort MDR counterfactual (Table S2b). Interpretive criteria also changed, since successive CLSI editions were applied as they came into force; the effect of holding breakpoints fixed is shown in Tables S5 and S6. Monthly denominators varied substantially, and each month contributed equally to the segmented models irrespective of the number of isolates tested (Table S3). The composition of the cohort by organism, specimen, and service also shifted across phases; an isolate-level model adjusted for these factors and clustered by patient preserved the pandemic-onset slope change, although the onset level shift was attenuated (Table S4). Antimicrobial consumption data for this hospital were not available for the study period, so the comparison with prescribing rests on national and regional sources rather than on institutional exposure.

The analytic approach carries limitations of its own. The counterfactual rests on an assumption that cannot be tested: that the pre-pandemic trend would have continued unchanged in the absence of the pandemic. Segmented linear regression was selected because its coefficients are directly interpretable as percentage points per month, but a linear model on a bounded proportion is not constrained to admissible values, and for three series the extrapolated pre-pandemic trend falls below zero at the primary contrast point; pPDR (−5.8%), MRSA (−5.0%), and *S. aureus* MDR (−0.2%). In January 2026, the same is true of five of the eight series, so that contrast is reported for completeness rather than as an estimate. For these three, the linear counterfactual overstates the departure, and the bounded refit below is the preferable estimate. Refitting on the logit scale with a quasi-binomial variance, which cannot generate inadmissible values, yields smaller differences in all three cases: +8.80 pp for pPDR (*p* < 0.0001), +9.06 pp for MRSA (*p* = 0.0598), and +9.03 pp for *S. aureus* MDR (*p* = 0.0777), so that the MRSA and *S. aureus* departures are not significant under the bounded specification (Table S3b). Where the extrapolations remain admissible, the two specifications are close in direction and magnitude; cohort MDR +24.27 against +22.38 pp, pXDR +8.93 against +8.85 pp, *E. coli* MDR +48.82 against +49.79 pp, *K. pneumoniae* MDR +42.21 against +35.64 pp, each significant under both, while VRE remains null under both (*p* = 0.6969 and *p* = 0.3830), so the principal findings do not depend on the choice of link. Phase boundaries were specified a priori rather than identified from the data, and the three phases differ substantially in length; shifting both interruption dates by up to 2 months in either direction gave counterfactuals between 19.37 and 24.27 pp, significant in every variant (all *p* ≤ 0.0002), with the pre-specified dates yielding the largest estimate (Table S1).

## 4. Materials and Methods

### 4.1. Study Design and Setting

This retrospective interrupted time-series study was conducted at the Antiguo Hospital Civil de Guadalajara “Fray Antonio Alcalde”, a tertiary-care referral hospital in Guadalajara, México. Isolate-level identification and antimicrobial susceptibility data for bacteria processed between October 2017 and January 2026, with valid identification and interpretable susceptibility results, were extracted from the clinical microbiology laboratory information system. The period was divided into three phases: pre-pandemic (October 2017 to February 2020), pandemic (March 2020 to February 2022), and post-peak (March 2022 to January 2026). The first interruption marked the onset of COVID-19 and hospital reorganization; the second coincided with México’s transition to the lowest national epidemiological-risk category and progressive hospital reconversion [34].

### 4.2. Isolate Selection and Deduplication

To limit overrepresentation of repeated cultures from the same episode, isolates were deduplicated by retaining the first isolate of each microorganism per patient within a rolling 14-day window, regardless of susceptibility profile; a later isolate of the same microorganism was eligible only if recovered more than 14 days after the previous eligible one. This follows the first-isolate principle used for cumulative antibiogram reporting, in which the susceptibility profile is deliberately not considered; admitting a later isolate because its profile had changed would preferentially retain isolates from treated patients and bias resistance proportions upward. Fungal and other non-bacterial isolates were excluded.

### 4.3. Identification and Antimicrobial Susceptibility Testing

Specimens were processed by routine diagnostic procedures. Colony suspensions were adjusted to 0.5 McFarland (VITEK DensiCHEK; bioMérieux, Marcy-l’Étoile, France) and identified on the VITEK 2XL platform with GN or GP cards. Susceptibility testing used organism-specific AST cards (AST-GP75, AST-P663, AST-N271, AST-N272, AST-N401, AST-N402, AST-N403) as available; the agents included on each card are listed in Table A1. Results were interpreted using the edition of the Clinical and Laboratory Standards Institute M100 standard in force at the time of testing [35], so that interpretive criteria followed successive editions across the study period rather than a single retrospective standard. The identification and susceptibility-testing platform was unchanged throughout the study period.

### 4.4. Resistance Phenotype Classification

Phenotypes were assigned per isolate using the organism-specific antimicrobial category sets of Magiorakos et al. [7]: 16 categories for Enterobacterales (*Enterobacteriaceae* in the source’s nomenclature), 8 for *Pseudomonas aeruginosa*, 9 for *Acinetobacter* spp., 16 of the 17 listed for *Staphylococcus aureus*, and 9 and 8 for *Enterococcus faecium* and the remaining enterococci, respectively. As the source requires, categories to which a species is intrinsically resistant were removed before the criteria were applied: penicillins for *Klebsiella* spp. and *Raoultella* spp.; penicillins, non-extended-spectrum cephalosporins, and cephamycins for the AmpC-producing genera (*Enterobacter* spp., *Citrobacter* spp., *Hafnia*, *Klebsiella aerogenes*, *Serratia* spp., *Morganella morganii*, and *Providencia* spp.), in which the penicillin plus β-lactamase inhibitor category was also restricted to piperacillin-tazobactam, with polymyxins removed for *Serratia* spp.; polymyxins, tetracyclines, and glycylcyclines for *Proteus* spp., *Morganella* spp., *and Providencia* spp.; streptogramins for *Enterococcus faecalis* and the other non-*faecium* enterococci; and glycopeptides for the *vanC* species *E. gallinarum* and *E. casseliflavus*. Carbapenems were not treated as an applicable category for any enterococcus. Organisms for which the source defines no category set (coagulase-negative staphylococci, streptococci, *Stenotrophomonas maltophilia*, other non-fermenting Gram-negative bacilli and fungi) and the taxa it places outside its scope (*Salmonella* spp., *Shigella* spp., *Streptococcus pneumoniae*, *Neisseria gonorrhoeae*) were non-evaluable and were excluded from every phenotype numerator and denominator.

MRSA was defined as *S. aureus* non-susceptible to oxacillin or cefoxitin. VRE were *E. faecalis*, *E. faecium*, or unspeciated enterococci resistant to vancomycin, excluding the intrinsically *vanC* species *E. gallinarum* and *E. casseliflavus*.

Resistant and intermediate results were treated as non-susceptible, and untested or uninterpretable results as missing. A category was evaluable when at least one applicable agent returned an interpretable result, and non-susceptible when at least one tested agent was non-susceptible; MDR was non-susceptibility in at least three categories. No isolate was tested against every category applicable to its organism, and the source states that under incomplete testing isolates, it can only be characterized as possible XDR or possible PDR, and that such results are not comparable with confirmed ones [7]. Both tiers are therefore reported as pXDR and pPDR throughout, assigned as follows: pXDR, an MDR isolate non-susceptible in all evaluable categories except at most two; pPDR, an MDR isolate with no susceptible result among the agents tested within its applicable categories.

### 4.5. Statistical Analysis

For each phenotype, the unit of analysis was the monthly proportion of phenotype-positive among evaluable isolates [100 × positive/(positive + negative)]; non-evaluable phenotype months were excluded from both numerator and denominator. Each series was modeled by segmented (interrupted) time-series regression, an established approach for estimating level and slope changes at pre-specified interruptions [36,37]:

*Yₜ = β_0_ + β_1_Tₜ + β_2_D2ₜ + β_3_D2ₜ(Tₜ − K_2_) + β_4_D3ₜ + β_5_D3ₜ(Tₜ − K_3_) + εₜ*

where Y_t_ is the monthly positivity (%); T_t_ is the number of months since study start; D2_t_ and D3_t_ indicate the pandemic and post-peak phases (1 from phase onset, 0 before); and K_2_, K_3_ are the months at which those phases begin. β_0_ and β_1_ give the pre-pandemic intercept and slope, β_2_ and β_3_ the level and slope change at the pandemic onset, and β_4_ and β_5_ at the post-peak onset. Net slopes were calculated as β_1_ (pre-pandemic), β_1_ + β_3_ (pandemic), and β_1_ + β_3_ + β_5_ (post-peak); models were fitted by ordinary least squares. Residual first-order autocorrelation was tested (Breusch–Godfrey) and, when significant (*p* < 0.05), refitted by generalized least squares with a first-order autoregressive error structure; the Durbin–Watson statistic and the estimated autoregressive coefficient (AR(1)φ) are reported for each model. Newey–West autocorrelation- and heteroscedasticity-consistent standard errors were also reported. Counterfactual contrasts—the difference between the fitted value and the extrapolated pre-pandemic trend—were reported in percentage points with 95% confidence intervals at the end of the pandemic phase (February 2022; primary) and at study end (January 2026). A joint Wald test of β_3_ + β_5_ = 0 assessed the combined slope change. The pre-pandemic slope is estimated 29 months, which is why February 2022, 24 months on, is the primary contrast.

Sensitivity analyses are reported in the Supplementary Materials: shifting both interruption dates by up to 2 months (Table S1); panel coverage by phase, and the cohort model refitted under progressively stricter coverage requirements (Table S2); weighted, binomial, and quasi-binomial specifications, with the February 2022 counterfactual refitted on the logit scale for every series (Table S3); an isolate-level segmented logistic model adjusted for species, specimen type, and service with robust standard errors clustered by patient, together with cohort composition by phase (Table S4); and non-susceptibility recomputed with breakpoints held fixed across the study period (Tables S5 and S6).

Antibiotic-class non-susceptibility was smoothed by locally weighted scatterplot smoothing (LOESS); a span of 0.3 was chosen because, in a 100-month series, it resolves change over roughly 30 months while suppressing month-to-month sampling variation, and the smoother is used for display only. Between-phase differences were compared by Mann–Whitney U on the monthly proportions, each month treated as the unit of observation, with Benjamini–Hochberg false-discovery-rate correction (adjusted values denoted q). Because consecutive monthly proportions are not independent, these class-level comparisons are descriptive and are not interpreted as tests of interruption. Analyses used Python 3.13.3 (pandas 2.2.3, NumPy 2.3.4, statsmodels 0.14.6, SciPy 1.16.0, Matplotlib 3.10.3); all tests were two-sided with *p* < 0.05 significant.

### 4.6. Ethics

This retrospective study used deidentified routine microbiological records and was approved by the Comité de Ética en Investigación of the Antiguo Hospital Civil de Guadalajara “Fray Antonio Alcalde” (approval number HCG/FAA/CEI-914/26), a committee registered with the Comisión Nacional de Bioética (registration validity reference no. 3018), in accordance with the Reglamento de la Ley General de Salud en Materia de Investigación para la Salud. Data extraction was performed in June 2026, after the study period closed and after the committee’s approval. The study was conducted in accordance with the Declaration of Helsinki.

## 5. Conclusions

Our principal findings show that the COVID-19 pandemic was associated with divergent changes in antimicrobial resistance at this Mexican referral hospital: an abrupt rise at pandemic onset followed by an incomplete return, concentrated in Gram-negative organisms, with *Escherichia coli* and *Klebsiella pneumoniae* departing from their pre-pandemic trajectories by different mechanisms and not returning to them. The pattern was mirrored at class level, where carbapenem and cephalosporin non-susceptibility returned to baseline, while fluoroquinolone non-susceptibility did not, in part reflecting a change in breakpoint. The highest burden of possible extensive and pandrug-resistant phenotypes occurred in the COVID unit and the intensive care unit. Aggregate measures risk both overstating a uniform pandemic resistance surge and masking the pathogens that matter most. Our analysis illustrates the value of organism-resolved, appropriately modelled surveillance, which distinguished pathogen-specific trajectories that aggregate analysis would have missed. Such surveillance, coupled with sustained antimicrobial stewardship, should inform preparedness for future health-system shocks. To our knowledge, this is one of the few studies in Mexico to characterize these resistance trajectories across the pre-pandemic, pandemic, and recovery periods using a long, continuous, organism-resolved time series.

## Figures and Tables

**Table 1:** Segmented regression model coefficients for MDR positivity in the full cohort.

Quantity	Estimate	SE	CI (−)	CI (+)	*p*-Value	NW–SE	NW-*p*-Value
β_0_ baseline level	60.09	2.21	55.71	64.48	<0.0001	1.37	<0.0001
β_1_ pre-pandemic slope	−0.60	0.12	−0.84	−0.35	<0.0001	0.11	<0.0001
β_2_ level shift March 2020	6.81	2.56	1.73	11.89	0.0092	2.54	0.0181
β_3_ slope change March 2020	0.73	0.19	0.34	1.11	0.0003	0.14	<0.0001
β_4_ level shift March 2022	−1.54	2.30	−6.11	3.03	0.5050	1.54	0.1822
β_5_ slope change March 2022	−0.36	0.15	−0.66	−0.05	0.0220	0.09	<0.0001
Net slope pre-pandemic	−0.60	0.12	−0.84	−0.35	<0.0001		
Net slope pandemic	0.13	0.14	−0.15	0.41	0.3488		
Net slope post-peak	−0.23	0.05	−0.33	−0.12	0.0001		
Counterfactual diff. Feb. 2022 (pp)	24.27	5.04	14.26	34.28	<0.0001		
Counterfactual diff. Jan. 2026 (pp)	40.14	10.32	19.64	60.63	0.0002		
Joint Wald (β_3_ + β_5_ = 0)	7.79				0.0064		
Durbin-Watson	1.23				0.0001		
AR (1)φ	0.38						
Model selected	GLS-AR (1)						

CI (−), lower limit of the 95% confidence interval; CI (+), upper limit of the 95% confidence interval; SE, standard error; NW-SE, Newey–West standard error; NW-*p*, Newey–West *p* value; AR (1)φ, first-order autoregressive coefficient; GLS, generalized least squares; pp, percentage points.

**Table 2:** Segmented regression model coefficients for MDR positivity in *Escherichia coli*.

Quantity	Estimate	SE	CI (−)	CI (+)	*p*-Value	NW-SE	NW-*p*-Value
β_0_ baseline level	88.03	3.41	81.27	94.79	<0.0001	3.46	<0.0001
β_1_ pre-pandemic slope	−1.15	0.18	−1.52	−0.79	<0.0001	0.22	<0.0001
β_2_ level shift March 2020	2.24	3.62	−4.94	9.43	0.5370	4.08	0.8766
β_3_ slope change March 2020	1.94	0.29	1.36	2.52	<0.0001	0.26	<0.0001
β_4_ level shift March 2022	−6.11	3.28	−12.63	0.41	0.0659	1.45	<0.0001
β_5_ slope change March 2022	−0.87	0.23	−1.31	−0.42	0.0002	0.11	<0.0001
Net slope pre-pandemic	−1.15	0.18	−1.52	−0.79	<0.0001		
Net slope pandemic	0.79	0.20	0.39	1.19	0.0002		
Net slope post-peak	−0.08	0.08	−0.24	0.08	0.3356		
Counterfactual diff. Feb. 2022 (pp)	48.82	7.59	33.75	63.88	<0.0001		
Counterfactual diff. Jan. 2026 (pp)	93.23	15.56	62.33	124.13	<0.0001		
Joint Wald (β_3_ + β_5_ = 0)	28.83				<0.0001		
Durbin-Watson	1.05				<0.0001		
AR (1)φ	0.47						
Model selected	GLS-AR (1)						

CI (−), lower limit of the 95% confidence interval; CI (+), upper limit of the 95% confidence interval; SE, standard error; NW-SE, Newey–West standard error; NW-*p*, Newey–West *p* value; AR (1)φ, first-order autoregressive coefficient; GLS, generalized least squares; pp, percentage points.

**Table 3:** Segmented regression model coefficients for MDR positivity in *Klebsiella pneumoniae*.

Quantity	Estimate	SE	CI (−)	CI (+)	*p*-Value	NW-SE	NW-*p*-Value
β_0_ baseline level	64.68	3.49	57.74	71.61	<0.0001	3.16	<0.0001
β_1_ pre-pandemic slope	−1.00	0.20	−1.40	−0.59	<0.0001	0.17	<0.0001
β_2_ level shift March 2020	14.46	5.09	4.35	24.56	0.0055	5.67	0.0108
β_3_ slope change March 2020	1.16	0.34	0.49	1.83	0.0009	0.44	0.0091
β_4_ level shift March 2022	−5.67	4.53	−14.67	3.32	0.2137	5.54	0.3060
β_5_ slope change March 2022	−0.05	0.29	−0.63	0.52	0.8496	0.38	0.8854
Net slope pre-pandemic	−1.00	0.20	−1.40	−0.59	<0.0001		
Net slope pandemic	0.16	0.27	−0.38	0.70	0.5530		
Net slope post-peak	0.11	0.10	−0.09	0.30	0.2840		
Counterfactual diff. Feb. 2022 (pp)	42.21	8.70	24.93	59.49	<0.0001		
Counterfactual diff. Jan. 2026 (pp)	88.31	17.56	53.44	123.19	<0.0001		
Joint Wald (β_3_ + β_5_ = 0)	23.78				<0.0001		
Durbin-Watson	1.64				0.0787		
AR (1)φ							
Model selected	OLS						

CI (−), lower limit of the 95% confidence interval; CI (+), upper limit of the 95% confidence interval; SE, standard error; NW-SE, Newey–West standard error; NW-*p*, Newey–West *p* value; AR (1)φ, first-order autoregressive coefficient; OLS, ordinary least squares; pp, percentage points.

**Table 4:** Antibiotic-class non-susceptibility across pandemic phases, selected classes and organisms.

Organism	Antibiotic Class	Pre-Pandemic (%)	Pandemic (%)	Post-Peak (%)	Pre-Pandemic vs Pandemic *p*-Value	Pre-Pandemic vs Pandemic *q*-Value	Pandemic vs Post-Peak *p*-Value	Pandemic vs Post-Peak *q*-Value
All organisms	Carbapenems	19.1	28.0	18.7	<0.0001	<0.0001	<0.0001	<0.0001
All organisms	Cephalosporins 3rd/4th	51.7	59.4	52.5	0.0002	0.0007	0.0001	0.0005
*Escherichia coli*	Fluoroquinolones	61.5	65.2	72.7	0.0030	0.0073	0.0004	0.0012
*Klebsiella pneumoniae*	Fluoroquinolones	37.6	46.5	49.3	0.0012	0.0034	0.1256	0.1919
*Staphylococcus aureus*	Fluoroquinolones	29.6	25.9	18.1	0.1299	0.1920	0.0005	0.0014
*Staphylococcus aureus*	Anti-staph β-lactam	25.6	21.5	12.9	0.0966	0.1603	0.0001	0.0004

Percentages are isolate-weighted mean non-susceptibility per phase (total non-susceptible/total tested). *p*, Mann–Whitney U on monthly proportions; *q*, Benjamini–Hochberg false-discovery-rate-adjusted *p* value.

## Data Availability

The data presented in this study are available on request from the corresponding author due to privacy and ethical restrictions, as the dataset derives from patient clinical microbiology records under applicable Mexican regulations.

## Supplementary Materials

The following supporting information can be downloaded at: https://www.mdpi.com/article/10.3390/antibiotics15101005/s1. Table S1: Alternative placement of the interruptions (cohort-wide MDR); Table S2: Panel coverage by phase; Table S3: Robustness of February 2022 counterfactual to model specification; Table S4: Case-mix adjustment (isolate-level segmented logistic regression, cohort-wide MDR); Table S5: Fixed-breakpoint sensitivity for agents revised during the study period (Enterobacterales); Table S6: Robustness of the composite MDR trajectory to fixed breakpoints (Enterobacterales).

## Abbreviations

The following abbreviations are used in this manuscript:

AMR	antimicrobial resistance
AR(1)	first-order autoregressive
AST	antimicrobial susceptibility testing
AWaRe	Access, Watch, Reserve (WHO antibiotic classification)
CI	confidence interval
CLSI	Clinical and Laboratory Standards Institute
COVID-19	coronavirus disease 2019
DDD	defined daily dose
ESBL	extended-spectrum β-lactamase
ESKAPE	*Enterococcus faecium*, *Staphylococcus aureus*, *Klebsiella pneumoniae*, *Acinetobacter baumannii*, *Pseudomonas aeruginosa* and *Enterobacter* spp.
ESKAPE-E	*Enterococcus faecium*, *Staphylococcus aureus*, *Klebsiella pneumoniae*, *Acinetobacter baumannii*, *Pseudomonas aeruginosa*, *Enterobacter* spp. and *Escherichia coli*
GLS	generalized least squares
GN/GP	Gram-negative/Gram-positive (VITEK identification card)
ICU	intensive care unit
IMSS	Instituto Mexicano del Seguro Social
LOESS	locally weighted scatterplot smoothing
MDR	multidrug-resistant
MRSA	methicillin-resistant *Staphylococcus aureus*
OLS	ordinary least squares
PDR	pandrug-resistant
pp	percentage points
pPDR	possible pandrug-resistant
PUCRA	Plan Universitario de Control de la Resistencia Antimicrobiana
pXDR	possible extensively drug-resistant
VRE	vancomycin-resistant enterococci
WHO	World Health Organization
XDR	extensively drug-resistant

## Appendix A

**Table A1:** Antimicrobial agents included on each VITEK AST card.

Antimicrobial Agent	Class	AST-GP75	AST-P663	AST-N271	AST-N272	AST-N401	AST-N402	AST-N403
Benzylpenicillin	Penicillins	○	●					
Ampicillin	Penicillins	○	●	●	○			
Oxacillin	Penicillins	●	●					
Ampicillin/sulbactam	β-lactam/β-lactamase inhibitor			●	●	●	●	●
Piperacillin/tazobactam	β-lactam/β-lactamase inhibitor			●	●	●	●	●
Cefazolin	Cephalosporins					○	●	
Ceftriaxone	Cephalosporins			●	●	○	●	
Ceftazidime	Cephalosporins				●	●	●	●
Cefepime	Cephalosporins			●	●	●	●	●
Cefoxitin (screen)	Cephalosporins	●	●					
Ceftaroline	Cephalosporins		●					
Ceftazidime/avibactam	Novel β-lactam combinations							●
Ceftolozane/tazobactam	Novel β-lactam combinations							●
Aztreonam	Monobactam			●	○			●
Ertapenem	Carbapenems			●	●	○	●	●
Imipenem	Carbapenems				●	●	●	●
Meropenem	Carbapenems			●	●	●	●	●
Doripenem	Carbapenems				●			
Amikacin	Aminoglycosides			●	●	●	●	●
Gentamicin	Aminoglycosides	●	○	●	●	●	●	
Tobramycin	Aminoglycosides			●	○			
Gentamicin (high-level, synergy)	Aminoglycosides	○	●					
Streptomycin (high-level, synergy)	Aminoglycosides	○	●					
Ciprofloxacin	Fluoroquinolones	●	●	●	●	●	●	●
Levofloxacin	Fluoroquinolones	●	●					
Moxifloxacin	Fluoroquinolones	●	○					
Erythromycin	Macrolide	●	●					
Clindamycin	Lincosamide	●	●					
Inducible clindamycin resistance (D-test)	Lincosamide	●	●					
Vancomycin	Glycopeptide	●	●					
Linezolid	Oxazolidinone	●	●					
Daptomycin	Lipopeptide	○	●					
Quinupristin/dalfopristin	Streptogramin	○						
Tetracycline	Tetracyclines	●	●					
Tigecycline	Glycylcycline	●	○	●	●	●	●	●
Colistin	Polymyxins				●	○	○	○
Fosfomycin	Fosfomycin				○	○	○	○
Nitrofurantoin	Nitrofuran	●	●	●	○	○	○	
Rifampicin	Ansamycin	●	●					
Trimethoprim/sulfamethoxazole	Folate-pathway inhibitor	●	●	●	○	●		
ESBL confirmation test	Phenotypic test			●	●	○	●	●

Filled circle (●), agent included on that AST card; open circle (○), agent used for a screen/confirmation result. AST, antimicrobial susceptibility testing.

**Table A2:** Segmented regression model coefficients for possible extensively drug-resistant (pXDR) positivity in the full cohort.

Quantity	Estimate	SE	CI (−)	CI (+)	*p*-Value	NW-SE	NW-*p*-Value
β_0_ baseline level	17.09	1.65	13.82	20.37	<0.0001	1.27	<0.0001
β_1_ pre-pandemic slope	−0.11	0.09	−0.30	0.07	0.2319	0.08	0.0962
β_2_ level shift Mar 2020	4.51	2.10	0.34	8.69	0.0342	2.42	0.0521
β_3_ slope change Mar 2020	0.18	0.15	−0.11	0.48	0.2177	0.13	0.0751
β_4_ level shift Mar 2022	−4.00	1.87	−7.71	−0.29	0.0351	1.72	0.0097
β_5_ slope change Mar 2022	−0.01	0.12	−0.25	0.23	0.9328	0.10	0.8435
Net slope pre-pandemic	−0.11	0.09	−0.30	0.07	0.2319		
Net slope pandemic	0.07	0.11	−0.15	0.30	0.5199		
Net slope post-peak	0.06	0.04	−0.02	0.15	0.1409		
Counterfactual diff. Feb 2022 (pp)	8.93	3.87	1.24	16.62	0.0233		
Counterfactual diff. Jan 2026 (pp)	13.11	7.92	−2.63	28.84	0.1016		
Joint Wald (β_3_+β_5_=0)	2.92				0.0909		
Durbin-Watson	1.54				0.0235		
AR (1)φ	0.23						
Model selected	GLS-AR (1)						

CI (-), lower limit of the 95% confidence interval; CI (+), upper limit of the 95% confidence interval; SE, standard error; NW-SE, Newey-West standard error; NW-*p*, Newey-West *p* value; AR (1)φ, first-order autoregressive coefficient; GLS, generalized least squares; pp, percentage points.

**Table A3:** Segmented regression model coefficients for possible pandrug-resistant (pPDR) positivity in the full cohort.

Quantity	Estimate	SE	CI (−)	CI (+)	*p*-Value	NW-SE	NW-*p*-Value
β_0_ baseline level	11.57	3.06	5.49	17.64	0.0003	1.52	<0.0001
β_1_ pre-pandemic slope	−0.33	0.15	−0.63	−0.03	0.0329	0.10	0.0082
β_2_ level shift Mar 2020	−0.37	2.07	−4.47	3.73	0.8587	3.36	0.9620
β_3_ slope change Mar 2020	0.66	0.24	0.19	1.12	0.0063	0.19	0.0027
β_4_ level shift Mar 2022	−2.71	1.95	−6.58	1.15	0.1663	1.80	0.0998
β_5_ slope change Mar 2022	−0.39	0.17	−0.71	−0.06	0.0217	0.15	0.0246
Net slope pre-pandemic	−0.33	0.15	−0.63	−0.03	0.0329		
Net slope pandemic	0.33	0.14	0.06	0.60	0.0184		
Net slope post-peak	−0.06	0.06	−0.18	0.07	0.3562		
Counterfactual diff. Feb 2022 (pp)	15.40	5.96	3.58	27.23	0.0113		
Counterfactual diff. Jan 2026 (pp)	25.45	12.52	0.58	50.32	0.0450		
Joint Wald (β_3_ + β_5_ = 0)	2.83				0.0959		
Durbin-Watson	0.61				<0.0001		
AR (1)φ	0.70						
Model selected	GLS-AR (1)						

CI (−), lower limit of the 95% confidence interval; CI (+), upper limit of the 95% confidence interval; SE, standard error; NW-SE, Newey-West standard error; NW-*p*, Newey-West *p* value; AR (1)φ, first-order autoregressive coefficient; GLS, generalized least squares; pp, percentage points.

**Table A4:** Segmented regression model coefficients for vancomycin-resistant enterococci (VRE) positivity.

Quantity	Estimate	SE	CI (−)	CI (+)	*p*-Value	NW-SE	NW-*p*-Value
β_0_ baseline level	7.51	5.72	−3.85	18.86	0.1924	3.74	0.1912
β_1_ pre-pandemic slope	0.13	0.31	−0.48	0.75	0.6669	0.33	0.3758
β_2_ level shift Mar 2020	−7.73	6.11	−19.86	4.40	0.2089	6.84	0.1045
β_3_ slope change Mar 2020	0.53	0.47	−0.42	1.47	0.2712	0.40	0.2448
β_4_ level shift Mar 2022	−7.88	5.46	−18.72	2.96	0.1522	3.27	0.0064
β_5_ slope change Mar 2022	−0.69	0.37	−1.41	0.04	0.0633	0.21	0.0003
Net slope pre-pandemic	0.13	0.31	−0.48	0.75	0.6669		
Net slope pandemic	0.66	0.33	0.00	1.32	0.0502		
Net slope post-peak	−0.03	0.13	−0.28	0.23	0.8305		
Counterfactual diff. Feb 2022 (pp)	4.89	12.51	−19.95	29.72	0.6969		
Counterfactual diff. Jan 2026 (pp)	−10.55	25.99	−62.16	41.06	0.6857		
Joint Wald (β_3_ + β_5_ = 0)	0.23				0.6310		
Durbin-Watson	1.22				0.0001		
AR (1)φ	0.40						
Model selected	GLS-AR (1)						

CI (−), lower limit of the 95% confidence interval; CI (+), upper limit of the 95% confidence interval; SE, standard error; NW-SE, Newey-West standard error; NW-*p*, Newey-West *p* value; AR (1)φ, first-order autoregressive coefficient; GLS, generalized least squares; pp, percentage points.

**Table A5:** Segmented regression model coefficients for methicillin-resistant *Staphylococcus aureus* (MRSA) positivity.

Quantity	Estimate	SE	CI (−)	CI (+)	*p*-Value	NW-SE	NW-*p*-Value
β_0_ baseline level	40.86	3.33	34.23	47.48	<0.0001	4.22	<0.0001
β_1_ pre-pandemic slope	−0.87	0.19	−1.24	−0.49	<0.0001	0.19	<0.0001
β_2_ level shift Mar 2020	9.67	4.59	0.56	18.79	0.0378	3.42	0.0046
β_3_ slope change Mar 2020	0.58	0.31	−0.03	1.20	0.0615	0.27	0.0289
β_4_ level shift Mar 2022	−2.65	4.06	−10.72	5.42	0.5156	3.59	0.4596
β_5_ slope change Mar 2022	0.15	0.26	−0.37	0.66	0.5716	0.20	0.4643
Net slope pre-pandemic	−0.87	0.19	−1.24	−0.49	<0.0001		
Net slope pandemic	−0.28	0.24	−0.76	0.20	0.2484		
Net slope post-peak	−0.14	0.09	−0.31	0.04	0.1298		
Counterfactual diff. Feb 2022 (pp)	23.68	8.02	7.76	39.60	0.0040		
Counterfactual diff. Jan 2026 (pp)	55.34	16.37	22.83	87.85	0.0011		
Joint Wald (β_3_ + β_5_ = 0)	12.06				0.0008		
Durbin-Watson	1.94				0.7717		
AR (1)φ							
Model selected	OLS						

CI (−), lower limit of the 95% confidence interval; CI (+), upper limit of the 95% confidence interval; SE, standard error; NW-SE, Newey-West standard error; NW-*p*, Newey-West *p* value; AR (1)φ, first-order autoregressive coefficient; OLS, ordinary least squares; pp, percentage points.

**Table A6:** Segmented regression model coefficients for multidrug-resistant (MDR) positivity in *Staphylococcus aureus*.

Quantity	Estimate	SE	CI (−)	CI (+)	*p*-Value	NW-SE	NW-*p*-Value
β_0_ baseline level	45.96	3.32	39.37	52.55	<0.0001	3.15	<0.0001
β_1_ pre-pandemic slope	−0.87	0.19	−1.25	−0.49	<0.0001	0.15	<0.0001
β_2_ level shift Mar 2020	11.02	4.57	1.95	20.10	0.0178	2.84	0.0001
β_3_ slope change Mar 2020	0.44	0.31	−0.17	1.05	0.1521	0.22	0.0454
β_4_ level shift Mar 2022	−1.46	4.04	−9.49	6.58	0.7196	2.65	0.5821
β_5_ slope change Mar 2022	0.24	0.26	−0.27	0.75	0.3489	0.15	0.1181
Net slope pre-pandemic	−0.87	0.19	−1.25	−0.49	<0.0001		
Net slope pandemic	−0.43	0.24	−0.91	0.05	0.0800		
Net slope post-peak	−0.19	0.09	−0.36	−0.01	0.0380		
Counterfactual diff. Feb 2022 (pp)	21.66	7.98	5.82	37.51	0.0079		
Counterfactual diff. Jan 2026 (pp)	52.40	16.30	20.04	84.76	0.0018		
Joint Wald (β_3_ + β_5_ = 0)	10.71				0.0015		
Durbin-Watson	2.18				0.3476		
AR (1)φ							
Model selected	OLS						

CI (−), lower limit of the 95% confidence interval; CI (+), upper limit of the 95% confidence interval; SE, standard error; NW-SE, Newey-West standard error; NW-*p*, Newey-West *p* value; AR (1)φ, first-order autoregressive coefficient; OLS, ordinary least squares; pp, percentage points.
