# Supplementary material for: Divergent Organism-Specific Trajectories of Multidrug Resistance Across the COVID-19 Pandemic: An Interrupted Time-Series Study in a Mexican Tertiary Hospital, 2017–Early 2026
Source: Antibiotics (Basel). 2026 Oct 10;15(10):1005. doi: 10.3390/antibiotics15101005 (PMC13700625; doi:10.3390/antibiotics15101005)
Supplement: Supplementary file 1 [file antibiotics-15-01005-s001.zip › antibiotics-4547922-supplementary.pdf]

Supplementary Materials

Divergent Organism-Specific Trajectories of Multidrug Resistance across the COVID-19 Pandemic: An Interrupted Time-Series Study in a Mexican Tertiary Hospital, 2017 to early 2026

These sensitivity analyses use the same cohort and segmented interrupted time-series model as the main text (first isolate per organism per patient within 14 days; non-mycology; October 2017 to January 2026, 100 months; 45,693 MDR-evaluable isolates). A percentage-point (pp) difference is the fitted level minus the extrapolated pre-pandemic trend, and the primary contrast is February 2022.

Table S1. Alternative placement of the interruptions (cohort-wide MDR).

The two interruptions were placed a priori in March 2020 (pandemic onset) and March 2022 (post-peak transition). To check that this placement does not drive the result, we refit the model with both knots shifted together, from two months earlier to two months later, keeping February 2022 as the point of comparison.

Interruption dates	Pandemic slope change (pp/month; p)	Post-peak slope change (pp/month; p)	Feb 2022 counterfactual (pp; p)
Jan 2020 / Jan 2022 (-2 months)	+0.90 (<0.001)	-0.62 (<0.001)	+21.4 (<0.001)
Feb 2020 / Feb 2022 (-1 month)	+0.82 (<0.001)	-0.52 (0.001)	+22.2 (<0.001)
Mar 2020 / Mar 2022 (primary)	+0.73 (<0.001)	-0.36 (0.022)	+24.3 (<0.001)
Apr 2020 / Apr 2022 (+1 month)	+0.68 (<0.001)	-0.41 (0.009)	+20.9 (<0.001)
May 2020 / May 2022 (+2 months)	+0.55 (0.004)	-0.31 (0.048)	+19.4 (<0.001)

Both slope changes keep their direction and significance across every placement, and the February 2022 excess stays positive and significant (+19.4 to +24.3 pp, all p < 0.001); the model the data selected, GLS-AR(1), was the same throughout. The primary MDR finding does not depend on exactly where the interruptions sit.

Table S2. Panel coverage by phase.

(Table S2a) Coverage per first isolate, in Magiorakos categories (the unit on which MDR is defined) and in individual agents with a valid interpretive result (S/I/R).

Phase	Categories tested (median)	Agents tested (median)	Evaluable first isolates (n)
Pre-pandemic	9	15	13,245
Pandemic	9	15	10,745
Post-peak	8	14	21,703

Testing breadth fell modestly over the study, from a median of 9 categories (15 agents) before the pandemic to 8 categories (14 agents) afterward. Because MDR requires non-susceptibility across at least three categories.

(Table S2b) The primary cohort MDR model refitted on isolates meeting progressively stricter panel-coverage thresholds. Complete-panel isolates (all applicable categories tested) were only 2.48% of the cohort, too few for a segmented model, so these graded restrictions are the feasible form of a comparable-coverage analysis.

Coverage restriction	n	% of cohort	Overall MDR	Feb 2022 counterfactual, pp (95% CI); p
All evaluable (primary)	45,693	100%	48.8%	+24.27 (14.26 to 34.28); <0.001
>= 5 categories tested	43,827	95.9%	50.1%	+24.33 (15.31 to 33.34); <0.001
>= 6 categories tested	41,121	90.0%	52.3%	+29.10 (19.65 to 38.54); <0.001
>= 7 categories tested	36,130	79.1%	53.3%	+20.54 (10.44 to 30.65); <0.001
>= 8 categories tested	28,435	62.2%	51.9%	+29.83 (15.15 to 44.52); <0.001
>= 60% of applicable	26,846	58.8%	46.0%	+27.41 (9.86 to 44.96); 0.003

Table S3. Robustness of February 2022 counterfactual to model specification.

(Table S3a) Cohort-wide MDR excess under alternative specifications (logit models back-transformed to the probability scale by the delta method).

Model	Excess (pp)	95% CI	p
Primary (GLS-AR(1), unweighted linear)	+24.27	14.26 to 34.28	<0.001
Weighted by monthly denominator (WLS)	+23.30	16.47 to 30.13	<0.001
Binomial segmented (logit link)	+22.38	18.41 to 26.35	<0.001
Quasi-binomial (logit, overdispersed)	+22.38	16.41 to 28.35	<0.001

The excess barely moves when months are weighted by their evaluable denominator or when a logit link replaces the linear-probability form. The monthly evaluable denominators were large and stable (165 to 601 isolates, median 458).

(Table S3b) Because a linear model on a bounded proportion can extrapolate outside 0 to 100%, the February 2022 counterfactual is also reported for every series under a quasi-binomial (logit-link) model. For the three series marked +, linear extrapolation is inadmissible, and the logit value is used in the main text.

Series	Linear (pp; p)	Quasi-binomial (pp; 95% CI)	p
MDR, whole cohort (Table 1)	+24.27 (<0.001)	+22.38 (16.41 to 28.35)	<0.001
pXDR, whole cohort (Table A2)	+8.93 (0.023)	+8.85 (4.07 to 13.63)	<0.001
pPDR, whole cohort (Table A3)[+]	+15.40 (0.011)	+8.80 (5.28 to 12.32)	<0.001
VRE (Table A4)	+4.89 (0.697)	-18.11 (-58.78 to 22.57)	0.383
MRSA (Table A5)[+]	+23.68 (0.004)	+9.06 (-0.37 to 18.48)	0.060
MDR, *E. coli* (Table 2)	+48.82 (<0.001)	+49.79 (41.95 to 57.62)	<0.001
MDR, *K. pneumoniae* (Table 3)	+42.21 (<0.001)	+35.64 (24.56 to 46.72)	<0.001
MDR, *S. aureus* (Table A6)[+]	+21.66 (0.008)	+9.03 (-1.00 to 19.06)	0.078

Table S4. Case-mix adjustment (isolate-level segmented logistic regression, cohort-wide MDR).

(Table S4a) Segmented logistic regression on evaluable first isolates (n = 45,693), with standard errors clustered by patient. The adjusted model adds the six most frequent species, specimen type, and clinical service; coefficients are on the log-odds scale.

Term	Unadjusted	Adjusted (species + specimen + service)
Pandemic-onset slope change	+0.0288 (<0.001)	+0.0376 (<0.001)
Post-peak slope change	-0.0149 (<0.001)	-0.0234 (<0.001)
Pandemic-onset level shift	+0.246 (<0.001)	+0.096 (0.179)

Adjusting for species, specimen type, and service left the pandemic-onset and post-peak slope changes essentially unchanged, and if anything, a shade stronger. The divergent MDR trajectory is therefore not explained by shifts in case-mix or specimen composition.

(Table S4b) Species composition of the MDR-evaluable cohort by phase (% of evaluable isolates in each phase) – the case-mix that the adjusted model in panel (a) accounts for.

Species	Pre-pandemic	Pandemic	Post-peak
Escherichia coli	33.83	30.13	33.48
Klebsiella pneumoniae	11.87	12.52	10.98
Pseudomonas aeruginosa	10.70	11.20	11.29
Acinetobacter baumannii complex	9.93	12.48	7.37
Staphylococcus aureus	9.23	9.00	10.60
Enterococcus faecalis	5.82	5.21	6.91
Other	18.63	19.47	19.37

Table S5. Fixed-breakpoint sensitivity for agents revised during the study period (*Enterobacterales*).

Percent non-susceptible by phase (pre-pandemic / pandemic / post-peak) among *Enterobacterales* (27,129 isolates), as reported (breakpoint in force at testing) and after applying a single fixed breakpoint to all isolates from the stored MICs, at the current and at an earlier in-window CLSI cutoff. Fixed susceptible cutoffs (MIC, ug/mL; non-susceptible = MIC above the cutoff): piperacillin-tazobactam 8 vs 16; amikacin 4 vs 16; gentamicin 2 vs 4; tobramycin 2 vs 4; ciprofloxacin 0.25 vs 1; levofloxacin 0.5 vs 2.

Agent	As reported (pre / pan / post)	Fixed current (pre / pan / post)	Fixed old (pre / pan / post)
Piperacillin-tazobactam	14.6 / 16.3 / 16.0	20.6 / 21.7 / 16.6	14.4 / 16.1 / 16.0
Amikacin	4.0 / 5.2 / 7.6	11.7 / 15.5 / 10.2	4.0 / 5.2 / 3.9
Gentamicin	34.0 / 34.4 / 28.8	34.3 / 34.9 / 28.7	33.7 / 34.1 / 28.2
Tobramycin	39.1 / 38.5 / 25.0	40.1 / 38.9 / 25.0	38.6 / 38.1 / 21.2
Ciprofloxacin	45.9 / 49.0 / 56.7	50.2 / 49.0 / 56.7	42.1 / 41.2 / 39.9

For gentamicin, tobramycin and piperacillin-tazobactam the phase pattern holds whatever breakpoint is used. For amikacin, part of the apparent post-peak rise reflects the 2023 aminoglycoside breakpoint lowering (M100-Ed33) and disappears under a fixed cutoff (7.6% to 3.9% post-peak).

Table S6. Robustness of the composite MDR trajectory to fixed breakpoints (*Enterobacterales*).

Segmented interrupted time-series for monthly *Enterobacterales* MDR, recomputed after reinterpreting every in-window revised agent (piperacillin-tazobactam, aminoglycosides and fluoroquinolones) from the stored MICs against a single fixed breakpoint, alongside the as-reported series. Slopes in percentage points per month; counterfactual = excess over the extrapolated pre-pandemic trend in February 2022.

Criterion	Pre-pandemic slope	Pandemic slope change	Post-peak slope change	Feb 2022 counterfactual
As reported	-0.90	+1.28	-0.44	+35.3 pp (<0.001)
Fixed current breakpoint	-0.89	+1.24	-0.42	+33.9 pp (<0.001)
Fixed old breakpoint	-1.00	+1.39	-0.55	+38.8 pp (<0.001)

The pandemic-onset rise and the post-peak fall hold under all three criteria, and the February 2022 excess stays large and highly significant (+33.9 to +38.8 pp, all $p < 0.001$). The primary MDR finding is therefore not an artifact of the CLSI breakpoint revisions that fell within the study period.
